# “HEGA”: the Basque version of the PaBiQ parental questionnaire, for clinicians and educators working in the Basque multilingual environment

**DOI:** 10.3389/fpsyg.2024.1211548

**Published:** 2024-04-10

**Authors:** Marie Pourquié, Urtzi Etxeberria, Hugues Lacroix, Philippe Prévost

**Affiliations:** ^1^Centre National de la Recherche Scientifique, IKER (UMR 5478), Bayonne, France; ^2^Université de Tours, INSERM, Imaging Brain & Neuropsychiatry iBraiN U1253, Tours, France

**Keywords:** multilingualism, speech and language therapy, parental questionnaire, language exposure, Basque

## Abstract

In this paper, we investigate the relevance of using a parental questionnaire (HEGA) to gather information on children’s language experience in Basque and early language development in order to better interpret language performance in that language. Both this questionnaire and use of language assessment in Basque are needed in the Basque Country, where multilingualism is well attested. The questionnaire was developed after the PaBiQ with additional questions meant to reflect the Basque context, notably its schooling linguistic model. The HEGA was administered to the parents of 186 bilingual children of the Northern Basque Country (age 4;2–9;1) whose language skills in Basque were assessed via a new test battery targeting different linguistic domains (HIGA). Several significant correlations were found between exposure to, and use of Basque and performance in lexical and morphosyntactic production and comprehension. Mixed-effect regression analyses revealed that language experience in Basque, and particularly the fact of being schooled entirely in Basque, were strong predictors of lexical and morphosyntactic outcomes. In contrast, phonological performance, as measured by nonword repetition, appeared to be less impacted by language experience in Basque. Finally, two children were identified as being at risk of language impairment, due to low language performance in Basque despite extended language experience. These results have important implications for clinicians and educators, in particular for detecting language difficulties in Basque-speaking bilingual children. They also show the need for assessing language abilities in Basque for children growing up in a solid Basque-speaking environment.

## 1 Introduction

Over the last 10 years or so, tremendous attention has been drawn to how best identify language impairment in bilingual children. This includes diagnosis of Developmental Language Disorder (DLD), a neurodevelopmental disorder involving persistent deficits in language that ‘are not explained by another neurodevelopmental disorder or a sensory impairment or neurological condition’ (ICD-11, World Health Organization, 2018). In many cases, language assessment tools are lacking in one or all languages of the child, and when language is assessed, the challenge is being able to disentangle low performance due to potential DLD or poor language experience, namely insufficiently long or rich exposure or use (Armon-Lotem et al., 2015).

The Basque Country is no exception to this situation. The 21st Century Basque society is typically multilingual, as Basque speakers also naturally acquire French and/or Spanish that are majority languages in the countries where Basque is spoken, i.e., France and Spain, in the Northern and Southern Basque Country, respectively.^1^ According to the Basque Government (2016) carried out by the Basque Government (Basque Autonomous Community, BAC), the Government of Navarre, and the Office Public de la Langue Basque, the number of Basque speakers has been increasing since the 1990s, which in 2016 amounted to 751,527 (28.4%) people aged over 16. This reflects an increase of 223,000 individuals since 1991, notably among young people (16–24), of whom 55% speak Basque.^2^ This trend is observed across the whole Basque Country, including the Northern Basque Country (NBC) in France, which has seen the largest increase of young Basque speakers compared to other age groups (19% in 2016 *vs* 11% in 1996). This increase results from the recovered prestige of the Basque language all over the territory and from language teaching policies, specifically the opening (officially in the 80s) of primary schools across the Basque Country, where teaching is carried out in Basque either partly (so-called bilingual schools) or entirely (immersion schools). In 2018–19, such schools received 40% of primary school enrolment in the NBC. The number of children enrolled in Basque schools has constantly been increasing since their creation. Importantly, while all Basque speakers are bilinguals (mainly with French or Spanish), there is a wide range of bilingual profiles across the Basque Country, with some speakers being more dominant in Basque than French or Spanish, others displaying the reverse pattern, and others having no language dominance.

Despite the evident bilingual nature of the Basque Country, language assessment of Basque-speaking children does not include Basque, due to a lack of (standardized) evaluation tools and defined developmental milestones in this language. Moreover, investigation of bilingual language development in Basque-speaking children has mainly focused on longitudinal case studies of Basque-Spanish pre-school age children (Elosegi, 1998; Larrañaga, 2000; Barreña, 2003) and on bigger data set collected from parental questionnaires – the Basque MB-CDI parental questionnaire^3^ (García et al., 2008, 2011, 2014; Barreña et al., 2008a,b; Ezeizabarrena et al., 2013). It has rarely addressed children growing up in the context of a neurodevelopmental disorder, including DLD. There is thus a lack of knowledge about language development in Basque-speaking children and about specific difficulties that Basque children with DLD may have (Pourquié, 2017).

When Basque is assessed in clinical contexts, evaluation is largely qualitative, based on spontaneous verbal interaction with the child and not in a normalized manner, i.e., by referring to norms on typically developing (bilingual) children. Instead of Basque, language assessment usually targets the other language of the child, typically French or Spanish, using (standardized) evaluation tools available in that language and the norms associated to those tools. Two main problems arise from this situation: first, since Basque speakers are for the vast majority – if not all – bilingual, using language assessment tools with monolingual norms is inadequate, with high risks of over- and underdiagnosis of language impairment (Thordardottir, 2015a). Second, using language evaluation tools created in French or Spanish to assess language in Basque-speaking children does not allow for assessment of Basque specific grammatical features. There are indeed major differences between Basque, which remains a language isolate with no known relatives and uncertain origins, and French and Spanish, which are both Romance languages. For instance, in what concerns grammatical features, Basque is a SOV language but French and Spanish are SVO languages; French and Spanish use prepositions that are free morphemes while Basque uses case marking corresponding to bound morphemes; Basque verbs agree with both subjects and objects while French and Spanish verbs only agree with subjects; relative clauses precede the noun in Basque while they follow it in French and Spanish; French and Spanish use clitics while Basque does not; etc. Further language evaluation in Basque would thus provide a more accurate picture of the children’s language abilities, which would lead to more appropriate language support. Testing children in Basque would also be feasible since many Speech-Language Therapists (SLTs) and educators in the Basque Country are bilingual.

While bilingual norms exist for some assessment tools (e.g., in German, Schulz and Tracy, 2011, and Lebanese Arabic, Zebib et al., 2017), this is the exception rather than the rule, including for French and Spanish. Many SLTs, especially those providing services to multilingual populations, call for norms on bilingual language development (Volpin et al., 2020). When such norms are not available, obtaining information on the language experience of the child, as well as his/her early language development is crucial (see Kašćelan et al., 2022). At minimum, this should provide SLTs and educators with information as to whether low language performance is obtained despite long and sustained experience in the language in question (which may be indicative of language difficulties or DLD), or whether it is accompanied with low language experience (which may not be indicative of language difficulties or DLD).

Parent questionnaires have been extensively used in research to gather background information, including evaluation of children’s language skills, and their adaptation to professional settings has been shown to be particularly relevant. Among the best-known parental questionnaires is the MacArthur-Bates Communicative Developmental Inventories (MB-CDI, Fenson et al., 1994), for which parents are asked to document their children’s lexical and grammatical abilities and gesture production. Significant correlations have been reported between the MB-CDI and direct language measures, showing the reliability of parents’ ratings (Feldman et al., 2005; Heilmann et al., 2005). Parent evaluation of their children’s language abilities has also been shown to be a strong predictor of DLD or language difficulties in children growing up in a monolingual setting (Callu et al., 2003; Surakka et al., 2023) or in a bilingual environment (Restrepo, 1998; Paradis et al., 2010). Parent assessment of their children’s language skills is particularly useful when one of the languages cannot be assessed directly.

Using a Basque version of the MB-CDI (Barreña et al., 2008a), studies on language development in Basque-speaking children have reported a significant impact of exposure to Basque on the development of lexical and morphosyntactic abilities. Barreña et al. (2008b) investigated 947 children aged 16 to 30 months. The sample was divided into three groups according to the percentage of Basque present in the children’s immediate environment (> 90%, 60–90%, and < 60%). In general, for both lexical knowledge and mean length of utterance, the group with the least exposure to Basque performed lower than the two other groups, especially as of age 27–28 months.

Previous research on language development in bilingual children has shown that different language domains may be impacted differently by language experience (see Unsworth, 2016 and Paradis, 2023 for overviews). For the lexicon, amount of exposure and socio-economic status (e.g., as measured by the mother’s education level) have been found to be particularly predictive of performance on both lexical production and comprehension (Cobo-Lewis et al., 2002; Golberg et al., 2008; Scheele et al., 2010). Likewise, quantity and quality of input can significantly influence performance and outcomes in morphosyntax, in production and comprehension, although the extent of this impact may differ across grammatical phenomena, owing, e.g., to their morphological or syntactic complexity and to the tasks being used (Paradis, 2010; Thomas et al., 2014; Thordardottir, 2015b).

In some studies, SES has been found to be a predictive factor of morphosyntactic outcomes as well (De Cat, 2021). Another aspect of quality of exposure that has drawn the attention of researchers concerns the proficiency level of the parents. In particular lower language performance (in lexicon and morphosyntax) by children has been found to correlate with lower degrees of nativeness of their parents in the language (Paradis and Jia, 2017; Unsworth et al., 2019). In Barreña et al.’s (2008b) study on Basque-speaking children, parents’ knowledge of Basque was also reported to affect the results: children with both parents speaking Basque were found to outperform those with only one parent speaking Basque for both lexicon and morphosyntax. In contrast, development of phonological skills in bilingual children seem to be less affected by language experience, especially when phonology is assessed via tools that control for lexical knowledge, such as nonword repetition tasks (Thordardottir, 2014; Dos Santos and Ferré, 2018).

One parental questionnaire now used in a variety of bilingual contexts is the Parents of Bilingual Children Questionnaire (PaBiQ, Tuller, 2015) developed during COST Action IS0804 (*Language Impairment in a Multilingual Society: Linguistic Patterns and the Road to Assessment,* 2009–2013).^4^ This questionnaire, available in 20 languages, documents variables known to impact bilingual language development, such as age of onset, quantity and quality of exposure, as well as early exposure (before the age of four). It also asks parents to evaluate the language skills of their children in all of his/her languages. Furthermore, a section is devoted to the child’s early history, such as the age of first word and age of first sentence, since delay in language emergence is observed in children with DLD (Rice et al., 2008; see also ICD-11, 2018), and whether the parents were concerned about language development in their children.^5^ The PaBiQ allows for the calculation of several composite scores and indexes about the risk of language impairment, early language exposure (before age 4), current language skills, and quantity and quality of current exposure and use, which can be used to better interpret language performance by the child.

In particular, studies using the PaBiQ have shown the relevance of the No risk index (and its component, the Positive early development index), which has been found to be a significant predictor of language performance across different language domains and in different bilingual settings. Based on stepwise multiple regression analyses on results from the PaBiQ and sentence and nonword repetition tasks administered to Bi-TD and Bi-DLD children in France and Germany, Tuller et al. (2018) found that the No risk index – and not the measures of language experience – was the main predictor, and often the only predictor of language performance, in both countries (see also Boerma and Blom, 2017).

Studies integrating bilingual children with DLD have found a differential impact of language exposure on morphosyntactic abilities compared to children with typical development (TD). De Almeida et al. (2017) investigated language skills in French in bilingual children (ages 5–8) with different first languages (Arabic, Portuguese and Turkish). After being tested in both of their languages via standardized tests, the bilingual children were divided into two groups, depending on whether they were deemed to be at risk of DLD (the Bi-DLD group) or not, i.e., showing Typical Development (the Bi-TD group). Using information collected from the PABIQ, significant correlations were found in the Bi-TD group between performance on a sentence repetition task and two composite scores of the PABIQ: use of French at home and language richness (in French). These correlations did not arise in the Bi-DLD group, suggesting that Bi-DLD children’s morphosyntactic skills did not improve as language experience increased (see also Armon-Lotem and Meir, 2016). Interestingly, no bilingualism variables, included the two that were reported to impact morphosyntactic performance, were found to significantly correlate with performance in nonword repetition.

To our knowledge no parental questionnaire is commonly used in the Basque Country for collecting information on the multilingual experience of Basque-speaking children. Some clinical, educational, and research centers use their own questionnaires (Anderson et al., 2019) and some questionnaires seem to be restricted to specific studies (e.g., Barreña et al., 2008b). Therefore, there is a need for the development of an easy-to-use parental questionnaire to be shared among the Basque community, in order to improve research, education and clinical practices adapted to the Basque multilingual environment.

In order to address the issue of the identification of atypical language development in Basque and provide adequate clinical services to Basque-speaking children with DLD, a parental questionnaire and a language assessment tool in Basque were developed by an interdisciplinary group of SLTs and researchers in psycholinguistics within the scope of the Nouveaux Commanditaires Sciences (NCS) program,^6^ which encourages a dialog between researchers and citizens, from a participative research perspective.

The aim of this paper is to present HEGA (*Haur Elebidunen Gurasoentzako Galdetegia* ‘Parental Questionnaire for Bilingual Children’), the Basque adaptation of the PABIQ questionnaire and its specificities, and to show its usability by clinicians, educators or researchers as a complementary tool to language assessment in Basque. In particular, this study sought to establish which measures of language experience correlate with, and predict, language skills in Basque.

We first hypothesized that the language skills (in Basque and French) estimated by the parents would be significantly correlated with the results on the different factors of language experience obtained throughout the questionnaire (early experience, length of exposure, language use, language richness, the parents’ proficiency in their languages, SES, and schooling model). The No risk index was also expected to impact language proficiency, as estimated by the parents.

We also hypothesized that language outcomes in Basque should be predicted by language experience in Basque (early exposure, language use at home, language richness and schooling model), with lesser impact on performance on phonology than on lexicon and morphosyntax. As to which measures from HEGA best predicted language skills and outcomes (in different language domains), this remained an open question, different predictors having been found in the literature, based on different methodological designs.

## 2 Materials and methods

### 2.1 Participants

HEGA questionnaires were completed by 186 parents of children enrolled in two types of schools in the NBC: Basque immersive schools where teaching is all in Basque (*n* = 136) and bilingual schools (*n* = 50) where half the teaching is in Basque and half in French. The children (88 boys and 98 girls) were aged 4;2 to 9;1 (*M* = 6;10, *SD* = 1;4) and had all received exposure to Basque and French. Fifteen of them had been exposed to another language, mainly Spanish (Spanish *n* = 8, English *n* = 5, Portuguese *n* = 1, and Wolof *n* = 1). Regarding age of exposure, 93/186 children (50%) were simultaneous Basque/French bilinguals and 77 (41.4%) were sequential bilinguals, including 40 who were exposed to the other language after age three. Among the 77 sequential bilinguals, 57 were first exposed to French (and in 33 cases exposure to Basque started after age three) and 20 were first exposed to Basque (and in seven cases, exposure to French started after age three). In the remaining 16 cases of our sample (8.6%), information about the age of first contact to Basque and/or French was missing. Regarding SES, all but eight children came from families where both parents had received post-secondary or university education, and only one child came from a family where both parents had received secondary education. Further information on the participants is presented in Section 3.1.

### 2.2 Materials

In order to address the issue of the identification of atypical language development in Basque and provide adequate clinical services to Basque-speaking children with DLD, as already mentioned above, the HEGA questionnaire and a language assessment tool in Basque named HIGA (*HIzkuntza Garapenaren Azterketa* ‘Language Development Assessment’) were developed by an interdisciplinary group of SLTs and researchers in psycholinguistics.

#### 2.2.1 HIGA: an oral language assessment tool in Basque

The HIGA assessment tool targets children aged 4–8 years. It has been normed on data collected from 254 children enrolled in immersive schools and percentile standards have been defined using the following scale: 95, 75, 50, 25 and 5. It contains 13 oral production and comprehension tasks targeting phonology, lexicon and morphosyntax (see Supplementary material 1). Five of them were selected for the present study in order to assess children’s phonological, lexical, and morphosyntactic abilities in production and comprehension: Non-word repetition, Object naming, Lexical recognition, Sentence production and Sentence comprehension. For homogeneity’s sake the other tasks were not analyzed. Following Tomblin et al.’s (1996) recommendations, children were considered to be at risk of having DLD when their language performance was low (below −1.25 SD) in at least two different domains.

##### 2.2.1.1 Object naming task

Object naming aims at assessing semantic knowledge and lexical access in production. Semantic knowledge corresponds to words’ meaning and lexical access to words’ phonological form retrieval. The task includes 32 items selected on the basis of their phonological structure (with or without coda) and their frequency. Five word types were established (see Supplementary material 2). The selected words had to have limited dialectal variability. For instance, the word *sagua* ‘mouse’ was included because it shows little variability in Basque; by comparison, the word *xinaurria* ‘ant’ was not selected as it can be said in different manners (*xinaurria, inurria, txindurria*, etc.). Color pictures depicting various objects, such as fruits, vegetables and animals are presented to the child (one at a time) who is instructed to say what the picture represents. If after 10 s the child has not produced the word, the examiner gives him/her a phonological cue in the form of the first sound of the target word. No other cue is allowed.

##### 2.2.1.2 Lexical recognition task

The lexical recognition task aims at assessing semantic knowledge and lexical access in comprehension. The task includes 16 nouns: 8 nouns taken from the Object naming task in order to assess whether some items that are not produced may nonetheless be understood, and 8 nouns related to various semantic categories and involving small dialectal variability. However, for four items two dialectal variants were considered as targets (*gauainara/xaguxarra* ‘bat’; *saskia/otarra* ‘basket’; *eskorga/karretila* ‘wheelbarrow’; *ganita/labana* ‘knife’). Each word is presented orally to the child, along with four pictures. The child is instructed to point to the picture corresponding to the oral stimulus. Among the four pictures, one is the target picture, one is the picture of a semantically related item (semantic distractor) and two depict unrelated items.

##### 2.2.1.3 Non-word repetition task

Non-word repetition (NWR) is generally used to assess phonological perception and production skills, and has been shown to be sensitive to DLD (Conti-Ramsden et al., 2001). The method followed to create the task items followed Ferré and dos Santos (2015). All segments used in the task are so-called language independent sounds, meaning that they are present in the majority of the languages of the world. Repetition of these segments should therefore be little impacted by language experience. The length of the items does not exceed three syllables, so as to minimize memory effects. Syllables are either simple (CV) or complex (i.e., involving a branching onset – CCV – or a coda – CVC). Moreover, in order to control lexical knowledge, it was made sure that no item resembled a real word in Basque, neither in standard Basque nor in any dialect. A total of 12 test items (see Supplementary material 3) are included, preceded by two training items. The task is based on color pictures depicting monsters, whose names correspond to the nonwords children are asked to repeat. All oral stimuli are pre-recorded.

##### 2.2.1.4 Sentence production and comprehension tasks

The HIGA morphosyntactic production and comprehension tasks focus on verb agreement with singular and plural subjects, direct objects and indirect objects. A total of seven inflected verb forms (verb auxiliaries) are tested twice for a total of 14 stimuli (see Supplementary material 4). The production task is a sentence completion task based on mini-scenes represented by two color pictures. The two pictures are quite similar but they differ in singular and plural agreement. The child is asked to describe all the pictures. If children have difficulties completing a sentence, examiners are allowed to provide the lexical verb, but not the auxiliary (in any form). The 14 test items of the task are preceded by one example.

The same 14 inflected verb forms are assessed in comprehension through a picture-selection task. This is a picture-sentence matching task in which children are asked to identify, from a group of four color pictures, the picture that best corresponds to a sentence presented orally. The four pictures are quite similar but differ in terms of verb agreement form or transitivity (see Supplementary material 5). Two training items are presented before the 14 test items. As in the NWR task, all oral stimuli are pre-recorded.

#### 2.2.2 The HEGA parental questionnaire

The HEGA questionnaire is a Basque adaptation of the PaBiQ. It was specifically designed to be used in clinical and educational contexts in the Basque Country, aiming to gather information on the child’s multilingual environment and his/her language developmental milestones.

The questionnaire is divided into nine sections for a total of 47 questions (see Table 1; Supplementary material 6 for the full list of questions). It exists in three versions (Basque, French, and Spanish) thus enabling a wide range of users to fill it in.

**Table 1:** Organization of the HEGA.

Section	Information collected	Number of questions
I. General information	Date and country of birth, country of residence, gender, languages currently spoken by the child, preferred language, number of siblings and position in siblings	11
II. Early language history (before age 4)	Age of the child’s first word and 1st sentence, parental concerns about the child’s language development, age of first contact with each language, exposure to each language before age 4	6
III. Current skills	Child’s proficiency in each language, as estimated by the parents	5
IV. Language use in the family	Languages used between the child and the parents, siblings, and other caretakers	6
V. Language richness	Languages used with friends and during specific activities, e.g., reading and watching TV	3
VI. Information about the parents	Birth country, language used at work, education and self-rated proficiency in each of their languages	6
VII. Language difficulties in the family	Difficulties concerning reading, spelling, speaking, and understanding	3
VIII. Educational information	Grade; schooling system; skip or repeat grade;	6
IX. Free comments	Any comments that the parents would like to share, either in general or on the questionnaire.	1

The first seven sections are similar to the PaBiQ (Tuller, 2015), while the eighth section was added to gather information on the child’s education (e.g., the school linguistic model the child was enrolled in), as this is very relevant to the Basque Country. Finally, the ninth and last section was added to gather parents’ free comments. All in all, a total of 11 questions were added to the original PaBiQ. Eight were taken from the Basque adaptation of the MacArthur-Bates CDI (García et al., 2014) and were added to the sections on general information (*n* = 4), language use in the family (*n* = 1), and information about the parents (*n* = 3). Moreover, following suggestions by the SLTs within the NCS action, one question was added to the section on language richness regarding the language in which the child is told stories (in addition to asking about the language in which the child reads), and one response choice was added to the question “Before age 4, did you worry about your child’s language?” in the early language history section. The answer “YES *after* age 3/4” was added to the original “YES/NO” answer. Finally, two questions about exposure to, and use of code-switching were added to the section on language use within the family, as this can provide relevant information regarding language experience in multilingual societies (Kašćelan et al., 2022).

As in the original PaBiQ questionnaire, different composite indexes and scores can be calculated: (1) a no risk index, (2) an early language exposure ratio (before age 4), (3) a parents’ estimate of their child’s current language skills score, (4) a score of language exposure and use at home, and (5) a score of language exposure and use with friends and during activities (also called language richness) (see Tuller, 2015). These indexes are explained below.

##### 2.2.2.1 No risk index

The no risk index brings together all the risk factors whose influence on the chances of a child having DLD is well-established: age of the early stages of acquisition (first words and first sentences), parental concern for the child’s language and existence of language difficulties within the family, with points associated with each answer (see Tuller, 2015). Three age range options are proposed in the questionnaire for age of first word (≤ 15 months, 16–24 months, and ≥ 25 months) and for age of first sentence (≤ 24 months, 25–30 months, and ≥ 31 months). In both cases, emergence of first word and first sentence in typical development corresponds to the first two options. Six points are associated with the first option, four with the second option, and none for the third option. Regarding parental concern, if none is expressed, an extra two points is added. Otherwise, no additional point is awarded. Finally, absence of language difficulties in the family (with respect to reading, understanding others, and expressing oneself) corresponds to 9 points. The maximum number of points is 23. Although the no risk index has proved to be sensitive to DLD, as seen above, the score below which concern should be raised as to a potential risk of language impairment is yet to be established. In Tuller et al. (2015), which involved bilingual children with or without DLD, all children with DLD had a no risk index score of 18 and below.^7^

##### 2.2.2.2 Early language exposure ratio (before age 4)

In Section 3 of HEGA, parents are asked to select the contexts in which their children were exposed to each of their languages before age 4 (e.g., with the mother, the father, the grandparents, a nanny, etc.). An overall number of contexts of language exposure is thus obtained, combining all contexts of exposure to all of the child’s languages. The early language exposure ratio is the percentage of contexts in which the child is exposed to a particular language with respect to the overall number of contexts of exposure.

##### 2.2.2.3 Parental estimation of current skills

This index combines, for each language, the scores of the five questions appearing in Section 4 of the questionnaire devoted to the children’s current language skills, as estimated by their parents. The answers are presented in a four-point Likert scale, which are associated to 0 to 3 points, the score of 3 corresponding to the highest (estimated) skills. The maximum number of points is 15. A score of 10 points (with five answers corresponding to ‘good’ language skills – 2 points) and above may be considered to be indicative of typical development for the language concerned.

##### 2.2.2.4 Score of language exposure and use at home

For each language of the child, parents are asked to rate the frequency of exchanges between the child and the mother, the father, the siblings, and any other caregiver (Section 5 of HEGA). Possible answers appear on a five-point Likert scale, ranging from 0 (never) to 4 (very often/always). The maximum score is 16 points.

##### 2.2.2.5 Language richness score (=score of language exposure and use with friends and during activities)

Language richness combines two sets of questions appearing in Section 6 of the questionnaire: (1) two questions about frequency of exchanges, for each language, between the child and his/her friends, and friends of the family (with answers presented on a five-point Likert scale, as above), and (2) four questions about frequency of language use during particular activities, i.e., reading, being read to, watching TV, and storytelling [with answers presented on a five-point Likert scale, ranging from 0 (never) to 4 (very often/always)]. The maximum score is 24 points.

### 2.3 Procedures and scoring

#### 2.3.1 General procedures

Eight schools in the NBC accepted to take part in the study: five Basque immersive schools and three French-Basque bilingual schools. Consent forms explaining the nature of each task and asking for permission to record data anonymously were obtained from all participating families. Testing always took place in a silent room at the child’s school during school hours. The examiner sat in front of the child and used the HIGA stimulus book to show the pictures, a computer to display the auditory stimuli in comprehension and repetition tasks, and a recording device to record the whole session. A total of 13 examiners participated in the data collection process. They were all members of the NCS group and were all familiar with the testing material as they actively participated in its design. Procedures, unanimously approved by the NCS group, were enforced concerning the application of a stop criterion (following 5 non answers in a row) and how many times an item could be presented in the NWR task (only once). Due to the COVID-19 sanitary crisis, the examiners and the children older than 6 years old were required to wear a mask. This did not, however, hamper the testing as the stimuli of the comprehension tasks were displayed from a computer. No unintelligible answers were reported due to the mask. The tasks were administered in a fixed order. At the end of the study, a present was sent to each school and addressed to the children and the staff that participated in the study.

The HEGA questionnaire was made available on-line to the parents through a Google form. In case some parents preferred to fill it in on paper, a printed version was made available. It was not possible to interview each family one by one due to the high number of participants and the COVID-19 crisis. This prevented us from checking that all the questions were answered and from providing help for questions that were felt to be unclear. Some space was left at the end of the questionnaire for parents to share comments or express what they had not understood. Eighteen parents left a comment. Only one complained that the survey was very long, and none reported any unclear question. In general, the parents used the comment section to share their multilingual experience, to explain the reason why they did not use Basque at home, to request Basque support for parents at school, and to explain the type of difficulty their child had (e.g., difficulty with pronunciation).

#### 2.3.2 Data scoring and analysis

In all the HIGA tasks that were used in this study, a correct answer was coded as 1 and an incorrect answer as 0. In the NWR task, a score of 1 corresponded to an item that was repeated identically as the stimulus. Otherwise, a score of 0 was awarded. In the Object naming task, correct answers that were produced spontaneously were scored as 1 and those for which help was provided were first coded as h1 and then scored as 1 (see Supplementary material 7 for examples). In the Sentence production task, production of inflected verb forms other than the expected one was counted as correct (so, as 1) in some specific cases: e.g., when the forms did not clash with the targeted tense (e.g., using the present progressive form *erortzen ari da/dira* ‘he/they are falling down’ for the present tense *erortzen da/dira* ‘he/they fall(s)’); when the forms corresponded to dialectal variants of the target forms too (e.g., *ematen dako* ‘(s)he gives it to him/her’ in Low Navarrese Basque used for *ematen dio* in Standard Basque).

Correlation analyses (controlled for age) were performed to explore the link between measures of language experience and language skills and outcomes. To model the relationship between accuracy in HIGA linguistic tasks (as indexed by the response variables from each task) and the potential predictors from the HEGA parental questionnaire, generalized linear mixed-effects regression analyses were performed. This kind of model can account for various predictors at the same time, for variance with either continuous or categorical predictors, and for random variation, using random effects. Taking into account random variation allowed us to control for sampling effects in our population (due to unbalanced groups of participants: 136 in immersive schools versus 50 in bilingual schools) and in our items (due to specific properties of each item). Therefore, in the models used in our analyses, Participant and Item are always included as random effects alongside the fixed effects. In all analyses, we tested whether the following variables were significant predictors of language performance: Age, Gender, Schooling Model, Length of Exposure in Basque, Total Basque used at home, Richness in Basque, Early exposure to Basque ratio (before age 4), No risk index, Positive early development index, Parental estimation of current skills in Basque, Mother’s education, and Father’s education.

## 3 Results

### 3.1 General results from the HEGA

General findings on the bilingualism variables documented by HEGA, for each language, appear in Table 2.

**Table 2:** General results (Mean and SD) from the main HEGA measures for Basque and French.

	Basque	French	*t*	*df^a^*	*p*
Age of first contact (months)	9.3 (15.6)	2.6 (9.7)	4.381	167	< 0.001
Frequency of early exposure (0–4 scale)	3.0 (1.1)	3.2 (1.1)	−1.248	183	0.214
Early contacts total (max. 8 pts)	4.6 (2.0)	4.6 (1.9)	0.145	175	0.885
Early language exposure ratio (before age 4)	48.9 (20.7)	49.6 (19.1)	−0.657	178	0.512
Length of exposure (months)	72.9 (21.0)	79.8 (19.4)	−4.381	167	< 0.001
Language used at home (max. 16 pts)	8.8 (4.9)	10.3 (5.0)	−2.124	183	0.035
Language richness (max. 24 pts)	10.6 (5.6)	13.8 (7.4)	−3.496	183	< 0.001
Proficiency level (mother) (0–4 scale)	2.2 (1.4)	3.8 (0.4)	−14.343	181	< 0.001
Proficiency level (father) (0–4 scale)	1.9 (1.5)	3.8 (0.5)	−16.293	181	< 0.001
Current skills (max. 15 pts)	9.3 (3.6)	9.7 (3.6)	−1.039	183	0.300

^a^*df* differed according to the number of parents who provided the expected information.

As can be seen, pairwise comparisons yielded significant differences between the two languages, including age of contact (earlier for French), and length of exposure (LoE), language use at home, language richness, and proficiency levels of the parents (all larger in French). In contrast, no difference between the two languages were found on variables targeting exposure during the first four years (early exposure, early contact – total, and percentage of exposure to Basque or French). No difference was found either between language skills in Basque and French as estimated by the parents.

However, these general results masked important differences in our population based on schooling type. Significant differences for all bilingualism variables were found between children enrolled in Basque immersion schools versus Basque/French bilingual schools, suggesting large variability in our sample (see Supplementary Table S1). Children enrolled in immersion schools had significantly wider language experience in Basque than children in bilingual schools. The reverse was found for French. In addition, parents of children in immersion schools tended to have significantly higher proficiency in Basque than parents of children in bilingual schools. Children did not significantly differ in terms of length of exposure to Basque, even though children in immersion schools had significantly earlier exposure to this language. Yet, children in bilingual schools tended to be significantly older (*M* = 90.0 months, *SD* = 11.3) than those in immersion schools (*M* = 79.4 months, *SD* = 17.6), accounting for similar LoE to Basque in each school system. It is important to note that no differences were found between the two groups regarding early developmental milestones and the no risk index (see Supplementary Table S2).

Finally, nineteen participants (17/136 in Basque immersive schools and 2/50 in French-Basque bilingual schools) had a low no risk index and could be considered to be at risk of DLD. Particular attention was paid to these children regarding the results on bilingualism variables and language performance presented below.

### 3.2 Analyses internal to the HEGA questionnaire

Given the wide age range of the child participants, partial correlation analyses (controlling for age) were performed between the estimated proficiency skills of the children, in both Basque and French, and measures of language experience and the No risk index.

As can be seen in Table 3, estimated proficiency measures significantly correlated with all measures of early exposure, LoE, Language used at home and Language richness, for both languages. For both Basque and French, Language used at home and Language richness yielded the strongest correlations (0.547 and 0.496, respectively, for Basque, and 0.626 and 0.586, respectively, for French). There were also significant correlations between the parents’ (self-rated) proficiency levels in each language and the estimated language skills of the children, with higher correlation coefficients observed for Basque. Note that the parents’ (self-rated) proficiency levels were also significantly correlated with Age of first contact, LoE, Language use at home, and Language richness, for Basque and French (see Supplementary material 8). For both the mother and the father, the highest correlation coefficients involved use of either language at home. In contrast, Table 3 shows that parent’s education did not strongly correlate with the children’s estimated language skills. One significant correlation was observed in Basque for Mother’s education, but it was low (0.186). Finally, there was a significant correlation between the measures of proficiency estimated by the parents in both Basque and French and the No risk index, with a higher correlation coefficient for French. Note that the correlation coefficient increased to 0.326 (*p* < 0.001) for Basque when Basque used at home and Basque richness were controlled for. For French, the correlation coefficient climbed to 0.367 (*p* < 0.001) with these two variables controlled for.

**Table 3:** Partial correlation analyses with current skills in Basque or French estimated by the parents (controlled for age).

	Basque		French	
	*r*	*p*	*r*	*p*
Age	−0.115	0.118	0.232	0.001
Age of first contact	−0.365	<0.001	−0.246	< 0.001
Frequency of early exposure (0–4 scale)	0.476	< 0.001	0.544	< 0.001
Early contacts total (max. 8 pts)	0.472	< 0.001	0.435	< 0.001
Early language exposure ratio (before age 4)	0.472	< 0.001	0.522	< 0.001
Length of exposure (months)	0.365	< 0.001	0.248	< 0.001
Language used at home (max. 16 pts)	0.547	< 0.001	0.626	< 0.001
Language richness (max. 16 pts)	0.496	< 0.001	0.586	< 0.001
Proficiency level (mother) (0–4 scale)	0.528	< 0.001	0.311	< 0.001
Proficiency level (father) (0–4 scale)	0.445	< 0.001	0.233	0.002
Education level (mother)	0.186	0.015	0.044	0.549
Education level (father)	0.061	0.412	0.027	0.715
No risk index (max. 23 pts)	0.189	0.013	0.353	< 0.001

Finally, we compared the children’s language skills, as estimated by the parents, according to schooling model. For Basque, language skills were estimated to be significantly higher for children in immersion schools than in bilingual schools [*t*(182) = 4.626, *p* < 0.001, Cohen’s d = 0.777]. The reverse obtained for language skills in French, with large effect sizes [*t*(182) = −5.126, *p* < 0.001, Cohen’s d = −0.861].

### 3.3 Analyses involving measures of the HEGA questionnaire and performance in the HIGA language tasks

In this section, we cross the data from the HEGA questionnaire with the performance on the five language tasks in Basque. We first report on correlation analyses between the HEGA measures and the language measures (Table 4). We then compare language performance in children enrolled in bilingual vs. immersion schools. Finally, we present the results of multiple regression analyses to identify predictors of language performance.

**Table 4:** Partial correlation analyses (controlled for age) between HEGA measures and performance in the five language tasks in Basque (Lexical production, Lexical comprehension, NWR, Morphosyntactic production, and Morphosyntactic comprehension).

	Lexical prod.		Lexical comp.		NWR		Morphosynt. prod.		Morphosynt. comp.	
	*r*	*p*	*r*	*p*	*r*	*p*	*r*	*p*	*r*	*p*
Age	−0.083	0.265	0.089	0.227	0.476	< 0.001	0.154	0.038	0.306	< 0.001
Age of first contact (Basque)	−0.426	< 0.001	−0.317	< 0.001	0.190	0.013	−0.394	< 0.001	−0.205	0.007
Frequency of early exposure (0–4 scale)	0.499	< 0.001	0.409	< 0.001	−0.135	0.066	0.483	< 0.001	0.267	< 0.001
Early contacts total (Basque) (max. 8 pts)	0.638	< 0.001	0.562	< 0.001	−0.088	0.239	0.480	< 0.001	0.282	< 0.001
Early exposure to Basque ratio (before age 4)	0.645	< 0.001	0.528	< 0.001	−0.134	0.071	0.497	< 0.001	0.279	< 0.001
Length of exposure (months)	0.426	< 0.001	0.317	< 0.001	−0.190	0.013	0.394	< 0.001	0.205	0.007
Basque used at home (max. 16 pts)	0.707	< 0.001	0.590	< 0.001	−0.154	0.038	0.547	< 0.001	0.274	< 0.001
Basque Richness (max. 24 pts)	0.717	< 0.001	0.522	< 0.001	−0.119	0.107	0.577	< 0.001	0.290	< 0.001
Proficiency level (mother) (0–4 scale)	0.603	< 0.001	0.520	< 0.001	−0.135	0.06	0.483	< 0.001	0.283	< 0.001
Proficiency level (father) (0–4 scale)	0.493	< 0.001	0.479	< 0.001	−0.043	0.565	0.454	< 0.001	0.252	< 0.001
Education level (mother)	−0.022	0.762	−0.003	0.973	−0.063	0.391	−0.033	0.659	−0.044	0.551
Education level (father)	0.158	0.033	0.108	0.147	−0.108	0.144	0.214	0.004	0.223	0.003
Total current skills (Basque) (max. 15 pts)	0.442	< 0.001	0.348	< 0.001	0.051	0.493	0.409	< 0.001	0.256	< 0.001
No risk index (max. 23 pts)	−0.029	0.705	0.040	0.605	0.254	< 0.001	−0.029	0.712	0.074	0.336

As can be seen in Table 4, mild to strong correlations were found between several HEGA measures and performance in Object naming (lexical production), Lexical recognition (lexical comprehension), Morphosyntactic production, and Morphosyntactic comprehension (with lower correlation coefficients for comprehension). Across the four tasks, Basque used at home, Basque richness, and early contacts in Basque yielded the strongest correlations. Proficiency level in Basque (for both parents) were also significantly correlated to performance on the four tasks. In contrast, Education level was mildly correlated with language performance on these tasks (for the father only). For NWR, fewer significant correlations were observed, and the coefficient correlations were lower than what was found for the other tasks. In particular, there was no significant correlation between NWR performance and Basque richness (*p* = 0.107), and significance was barely reached with Basque used at home (*p* = 0.038). Age was the strongest variable with which NWR performance was significantly correlated (*r* = 0.476). The score for current language skills in Basque as estimated by the parents significantly correlated with all individual responses (moderately so with Morphosyntactic comprehension, *r* = 0.256), except for NWR (*p* = 0.493). As to the No risk index, the only significant correlation was found with the performance on NWR, but here again, the correlation coefficient was moderately high (*r* < 0.300).

Finally, the schooling model was found to affect language performance. Children in immersion schools performed significantly better than children in bilingual schools in all tasks [Object naming: *t*(181) = 15.138, *p* < 0.001; Lexical recognition: *t*(181) = 7.023, *p* < 0.001; NWR: *t*(181) = −3.426, *p* < 0.001; Morphosyntactic production: *t*(179) = 9.249, *p* < 0.001; Morphosyntactic comprehension: *t*(180) = 5.718, *p* < 0.001]. Scattered plots for all analyses detailed above can be found in the Supplementary material.

We now turn to multi-regression analyses. All the potential predictors (see Section 2.3.2) were included for all tasks using stepwise regression, but only the most relevant regression model is reported.

#### 3.3.1 Object naming

The variables related to Basque exposure had a significant impact on the performance at Object naming. In particular, as can be seen in Table 5, the schooling model (with Basque only used as the baseline) negatively predicted accuracy, meaning that children in French-Basque bilingual schools scored significantly lower than the children in immersive Basque schools. In contrast, LoE to Basque and Use of Basque at home positively predicted accuracy. Finally, the level of education of both parents was also predictive of accuracy on the Object naming task, but only significantly so for the father. Looking further at the data, we found that there was more variation in the father’s educational level, with the mother’s level being generally higher.

**Table 5:** Regression analysis on HEGA measures and performance in object naming (lexical production).

Term	Estimate	SE	z value	*p*-value
(Intercept)	−3.82	1.357	−2.814	0.005
Schooling model (Bilingual Basque/French)	−2.309	0.276	−8.358	< 0.001
Length of exposure (Basque)	0.015	0.005	2.83	0.005
Basque used at home (max. 16 points)	0.182	0.029	6.383	< 0.001
Education level (mother)	0.48	0.289	1.66	0.097
Education level (father)	0.379	0.168	2.251	0.024

#### 3.3.2 Lexical recognition

As was the case for Object naming, the schooling model (with Basque only used as the baseline) negatively predicted accuracy on lexical recognition, while Use of Basque at home was a positive predictor of performance on this task (see Table 6). No other predictors were identified for the performance on lexical recognition.

**Table 6:** Regression analysis on HEGA measures and performance in lexical recognition (lexical comprehension).

Term	Estimate	SE	z value	P-value
(Intercept)	1.205	0.367	3.279	0.001
Schooling model (Bilingual Basque/French)	−0.711	0.222	−3.198	0.001
Basque used at home (max. 16 points)	0.121	0.024	5.116	< 0.001

#### 3.3.3 Non-word repetition

For the NWR task, none of the tested variable, except for Basque use at home, significantly predicted performance (see Table 7). The schooling model and the No risk index had a significant impact on the Akaike information criterion (AIC) index of the model, but alone they were not significant.

**Table 7:** Regression analysis on HEGA measures and performance in NWR.

Term	Estimate	SE	z value	P-value
(Intercept)	1.481	0.974	1.52	0.13
Schooling model (Bilingual Basque/French)	0.364	0.318	1.144	0.25
Basque used at home (max. 16 points)	−0.093	0.03	−3.153	0.002
No risk index (max. 23 points)	0.077	0.042	1.823	0.068

#### 3.3.4 Sentence production

The schooling model also had a significantly negative effect on performance in Sentence production (Table 8), with individual responses in the French-Basque bilingual school group being significantly lower than in the immersive Basque school group. The total amount of Basque used at home and the no risk index also predicted performance significantly. As to the father’s educational level, although its impact was not statistically significant, it improved the fit of the model according to the AIC.

**Table 8:** Regression analysis on HEGA measures and performance in sentence production.

Term	Estimate	SE	z value	P-value
(Intercept)	−4.679	2.033	−2.302	0.021
Schooling model (Bilingual Basque/French)	−2.411	0.484	−4.986	< 0.001
Basque used at home (max. 16 points)	0.166	0.047	3.506	< 0.001
No risk index (max. 23 points)	0.139	0.074	1.893	0.058
Education level (father)	0.565	0.309	1.826	0.068

#### 3.3.5 Sentence comprehension task

As seen for all the other language measures, children in Basque immersion schools had significantly higher performance on Sentence comprehension than children attending bilingual schools (Table 9). Age and the father’s education level also had a significant (and positive) impact on Sentence comprehension.

**Table 9:** Regression analysis on HEGA measures and performance in sentence comprehension.

Term	Estimate	SE	z value	P-value
(Intercept)	−2.632	0.934	−2.818	0.005
Age (in years)	0.544	0.091	5.98	< 0.001
Schooling model (Bilingual Basque/French)	−1.831	0.262	−6.984	< 0.001
Education level (father)	0.433	0.177	2.446	0.014

We end this section with some findings on the 19 children with a low No risk index, ranging from 9 to 17 (out of 23). For 14 of these children, the parents estimated their language skills in Basque to be low (below 10 out of 15), including eight with low skills in the other language as well. Using Tomblin et al.’s (1996) recommendation for identifying DLD (see above), we found that five of these 19 children had language performance below −1.25 SD in at least two different domains. However, these children were among the youngest in our sample (younger than 4;6). In three cases, experience with Basque during the first four years was low (e.g., fewer than 50% Basque exposure). For the two other children, exposure to Basque was much higher, and scores for Basque use at home and Basque richness were at ceiling, which could be cause for concern. For the other children whose skills were rated low by their parents, language performance was above −1.25 SD in at least four of the tasks.

## 4 Discussion and conclusion

In this paper, we investigated the relevance of using a parental questionnaire (HEGA) to gather information on children’s language experience in Basque and early language development in order to better interpret language performance in that language. Both this questionnaire and use of language assessment in Basque are needed in the Basque Country, where multilingualism is well attested. The questionnaire was developed after the PaBiQ (Tuller, 2015) with additional questions meant to reflect the Basque context, notably its schooling linguistic model. The language tasks came from a new language battery in Basque targeting different linguistic domains (HIGA). A total of 186 children of the NBC (age 4–9) and their parents participated in the study.

As hypothesized, significant correlations were found between several measures of bilingualism factors and the language skills of the children, as estimated by their parents or assessed via language tasks (except for NWR, see below). High correlations were particularly observed with language use at home and richness, which confirms what has been reported in the literature. Parent proficiency also correlated with estimated language skills and all individual responses, which is akin to recent findings pointing to the importance of the quality of language exposure for language development (see Paradis, 2023). These results also confirm what has been found for Basque on younger children regarding the impact of exposure to Basque and parent proficiency in Basque on language abilities (Barreña et al., 2008b). Language experience played a lesser role for NWR, with lower correlation coefficients than for all other language measures, which confirms what has been reported in the literature (Thordardottir, 2014; de Almeida et al., 2017). During the development of the NWR task of the HIGA, particular care had been paid to making the items as less word-like as possible, which included taking into account different Basque dialects. Lexical knowledge was thus well controlled for this task, which can explain the very low impact of language experience on the results. In contrast to measures of language experience, few significant correlations were found between the no risk index and language measures. The only ones involved parent ratings of language skills and NWR performance. Note, however, that some of the children with a low no risk index and low individual language performance were quite young (below 4;6 years) with little Basque experience, thus preventing any conclusion about a potential language disorder. It should also be noted that the studies that reported on a significant impact of the no risk index on language performance all involved a group of TD children and a group of children with DLD, in contrast to our study. Moreover, some parents may have had difficulties answering some of the questions directly impacting on the calculation of the no risk index, such as age of first words and age of first sentences. These questions have been identified as particularly complicated for some parents (i.e., what should be considered as a word or a combination of words may not appear to be very transparent for many), which is the reason why some authors advocate for a person-to-person administration of questionnaires such as the PaBiQ (Tuller, 2015). However, due to the pandemic, the parents filled in the questionnaire by themselves on-line, and did not benefit from any assistance. This notwithstanding, two of the young children with a low no risk index in our study had high ratings for Basque use at home and Basque richness, which may be cause for concern. A clear research perspective involves the recruitment of French/Basque children that receive speech-language therapy in order to investigate the effect of language experience in their language performance in Basque, as well as the effect of the no risk index.

As announced in the introduction, an open question remained as to which measures from HEGA could best predict language skills and outcomes in different language domains because different predictors were found in the literature based on different methodological designs. Of all the predictors investigated in this study, the schooling system in which the children were enrolled came out systematically, with children attending immersion schools outperforming children in bilingual schools in all language measures. Large and significant differences between the two school models were found with respect to many measures of language experience in our study, including use of Basque at home, richness in Basque, exposure to Basque until age four, and parent proficiency in Basque. We take the results of the regression analyses to reflect this difference. In short, it is the combination of the different measures of language experience, which comes out as the main predictor of language performance. Among the other potential predictors, SES, as measured by parent education, only played a significant role in the results for Object naming (lexical production), which has been widely reported in the literature. It was not identified as a major predictor for the other language tasks (including lexical comprehension) and for the language skills as estimated by the parents. This could be explained by the little variability in SES in our population sample, which mainly consisted of individuals with post-secondary and university education. Further studies on language development in Basque-speaking children should be more inclusive, expanding recruitment to more under-privileged communities.

Finally, we found that performance in production tended to be impacted by language experience to a larger extent than comprehension. Studies have reported that bilinguals, as compared to monolinguals, present a larger gap between production and comprehension abilities, in both their languages, with comprehension typically surpassing production. This asymmetry is commonly called the ‘expressive-receptive gap’ (Gibson et al., 2014) and has been attributed to a weakness in lexical-semantic links, which has a stronger impact on production than on comprehension and is highly influenced by language exposure (Gollan et al., 2008; Keller et al., 2015). This would explain why Object naming and Sentence production are particularly affected by language exposure in our study. Another factor that might contribute to the expressive-receptive gap is linguistic typology. Anderson et al. (2019) presented evidence for such a gap in the grammatical abilities of school-aged Basque-Spanish bilingual children and found it to be wider regarding grammatical structures that are not shared between the languages of the child. This proposal would merit to be investigated more thoroughly, for example by comparing bilinguals with a combination of languages that are either typologically related or unrelated, and with a comparable amount of language experience in the L2.

In short, the present study has shown that the parental questionnaire HEGA is a useful tool as a complement of language assessment in Basque, allowing better interpretation of children’s linguistic abilities by taking into account their multilingual environment. More specifically, it was shown that language experience in Basque, and particularly the fact of being schooled entirely in Basque, was the best predictor of lexical and morphosyntactic outcomes. In contrast, phonological skills appeared to be less impacted by exposure to, and use of Basque. Finally, two children were identified as being at risk of language impairment, which further shows that crossing information from the HEGA questionnaire and the HIGA tools can be particularly useful for identifying potential language disorders that would be the manifestation of underlying developmental deficits.

The results of the present study have important implications for clinicians and educators. First, the information provided by parents is coherent, suggesting that they can be taken into account when a decision has to be made about whether a child is in need of specific support or not. The HEGA also appears to be user-friendly, as shown by the absence of negative comments regarding its length or the complexity of some of its questions. Second, the fact that measures of language experience in Basque may impact on language performance means that language scores should not be considered on their own; they should also be put in perspective with information related to the linguistic environment of the child, which goes in the same direction as findings on language assessment in bilingual children growing up in different multilingual contexts (Armon-Lotem et al., 2015). Including a subpart on the schooling system (immersive or bilingual) into the questionnaire proved to be crucial in this respect. Regarding children from immersive schools, whose number is steadily increasing these last years (from 2004 to 2021, there was an 86% increase in the number of children receiving immersive teaching in the immersive schools; OPLB 2021), the information obtained from HEGA reveals that they tend to be more exposed to Basque than to French. Therefore, it is absolutely necessary that SLTs be able to assess the language skills of these children with language tasks in Basque, which should lead to more reliable diagnosis. As SLTs opt for assessing children from bilingual schools in French rather than in Basque because French is mostly their dominant language, the reverse, i.e., assessing children from immersive schools in Basque rather than in French, only seems natural. Third, our results show that performance on NWR, being less impacted by language experience, is particularly useful for identifying language disorder in multilingual contexts. As to children’s performance in vocabulary and morphosyntax, it should be interpreted along with the information collected from HEGA, especially Basque use at home and Basque richness. This is a very important message to convey to professionals in need of assessing language in bilingual children, given the challenge that they face. The next step is to conduct a study involving SLTs and educators to investigate the usefulness of HEGA in their daily practice for identifying children with DLD or in need of language support.

## Editor’s note

Maria-José Ezeizabarrena edited the article in collaboration with Melita Kovacevic, University of Zagreb, Zagreb, Croatia.

## Data availability statement

The raw data supporting the conclusions of this article will be made available by the authors, without undue reservation.

## Author contributions

UE participated in the Basque adaptation of the PaBiQ questionnaire, wrote the introduction, and took part in discussions regarding the results of the study. HL participated in the Basque adaptation of the PaBiQ questionnaire (Spanish version in particular), and running all the analyses of the paper, creating graphs, and wrote the methods and results sections. PP took part in discussions regarding the results of the study, wrote all the parts of the manuscript (introduction, methods, results, and discussion), and shared his knowledge on the French PaBiq and his experience within the LITMUS community. MP took the initiative to collaborate with researchers and SLTs to adapt the PabiQ for Basque in order to gather information on the multilingual environment of the participants that would be tested on the HIGA oral language assessment tool, described the materials, she and her colleagues have developed within the scope of the program Nouveaux Commanditaires Sciences along with a dozen Speech and Language Therapists, and wrote all the parts of the manuscript (introduction, methods, results, and discussion). All authors contributed to the article and approved the submitted version.

## Footnotes

^1^Basque is not only spoken in the Basque Country, but also in various Basque communities around the world, in the so-called Basque diaspora, i.e., people of Basque origin living outside the borders of the Basque Country. We will not consider these communities here.

^2^A new survey containing data collected in 2021 was published in 2023. For the moment, only the data from the BAC is available, i.e., not counting the data from the NBC and Navarre, and they continue showing an increase of Basque speakers in people aged over 16 years. According to this new survey (2021eko Inkesta Soziolinguistikoa) 36.2% of the population of the BAC speaks Basque, which corresponds to 680.629 people, i.e., about 50.000 more than in 2016.

^3^Notice that there are five CDI instruments adapted to Basque: long CDI-1 & CDI-2 (Barreña et al., 2008a); Short CDI-1 & CDI-2 (García et al., 2008, 2011) and CDI-3 (García et al., 2014).

^4^http://www.bi-sli.org/

^5^The PaBiQ was originally inspired by two parental questionnaires, the Alberta Language and Development Questionnaire (ALDeQ; Paradis et al., 2010), focusing on variables related to bilingualism, and the Alberta Language Environment Questionnaire (ALEQ; Paradis, 2011), which documents L1 development and risk factors of language impairment.

^6^https://www.joursavenir.org/activities/ncs/en

^7^Note that in contrast to the PaBiQ, an additional option (“I do not know”) was inserted as an answer to the questions targeting age of first word and first sentence in the HEGA questionnaire, as some parents may find it difficult to answer. When the parents chose the “I do not know” option for both questions, the no risk index was not calculated (14 cases). When the “I do not know” option was selected for one of the two questions, it was decided that some points should be awarded if the answers to the other two questions (age of 1st sentence/word and parental concerns) were congruent. In particular, 6 or 4 points were assigned if the age of 1st word/sentence was deemed to be typical (i.e., not appearing after 25 and 31 months respectively) AND parents expressed no concerns (22 cases). Zero points were assigned if the age of 1st word/sentence was above these cut-offs AND parents expressed concerns (2 cases). When incongruency was observed in the answers of the two questions (e.g., 1st word/sentence above 25 or 31 months AND no parental concerns), no adjustment was made and the no risk index was not calculated (2 cases). Note also that when parents answered positively to the answer about parental concerns *after* age 3 or 4, which was added to the original PaBiQ, it was decided to attribute the same score as when they answered positively to the question about concerns *before* age 3 or 4 present in the original PaBiQ (i.e., 0 points).
