# Supplementary material for: “HEGA”: the Basque version of the PaBiQ parental questionnaire, for clinicians and educators working in the Basque multilingual environment
Source: Front Psychol. 2024 Apr 10;15:1211548. doi: 10.3389/fpsyg.2024.1211548 (PMC11042248; doi:10.3389/fpsyg.2024.1211548)
Supplement: Supplementary file 1 [file Data_Sheet_1.pdf]

Supplementary material

Supplementary material 1

Composition of HIGA: oral language assessment tool for Basque-speaking children aged 4-8

N° Task	Type of task	Number of stimuli
1	Object naming	32
2	Production of adjectives	8
3	Action naming	6
4	Sound perception	10
5	Non-word repetition task	12
6	Lexical recognition	16
7	Body-part naming and comprehension	8
8	Colour naming and comprehension	6
9	Topology naming and comprehension	8
10	Sentence production	14
11	Sentence comprehension	14
12	Case morphology production	10
13	Negative clause production	5

Supplementary material 2

Word types assessed in the HIGA Object naming task of HIGA

Word type	Syllable type	N syllables	N items	Example
Short-simple	CV or V	< 3 syllables	6	/ʃagu/ 'mouse'
Short-complex	**VC** or CVC	< 3 syllables	6	/**as**to/ 'donkey'
Long-simple	CV or V	> 3 syllables	6	/liburu/ 'book'
Long-complex	VC or **CVC**	> 3 syllables	6	/**ilar**gi/ 'moon'
Less frequent	CV, V, VC and CVC	≤ 3 syllables	8	/labe/ 'oven'

Supplementary material 3

Stimuli used in the Non-word repetition task of HIGA

Item	Complex(c)/ Simple(s) onset	With+(out-) coda	N° syllables
/adɔ/	s	-	2
/sapɔn/	s	+	2
/lɔpaʀ/	s	+	2
/ki**bʀ**a/	c	-	2
/**fl**enis/	c	+	2
/mɔʃɛk/	s	+	2
/salimut/	s	+	3
/**gʀe**lanta/	c	-	3
/kabula/	s	-	3
/ɔɲustɔ/	s	+	3
/parasɛk/	s	+	3
/i**tʀ**eko/	c	-	3

Supplementary material 4

Basque verb auxiliary types assessed in both Sentence production and comprehension tasks of HIGA

Inflected form (verb auxiliary)	Transitivity	SG/PL subject	SG/PL direct object	SG/PL indirect object
da	intransitive	3rd SG		
dira	intransitive	3rd PL		
du	transitive	3rd SG	3rd SG	
dute	transitive	3rd PL	3rd SG	
ditu	transitive	3rd SG	3rd PL	
dio	ditransitive	3rd SG	3rd SG	3rd SG
die	ditransitive	3rd SG	3rd SG	3rd PL

Supplementary material 5

Sentence production task of HIGA: picture set examples

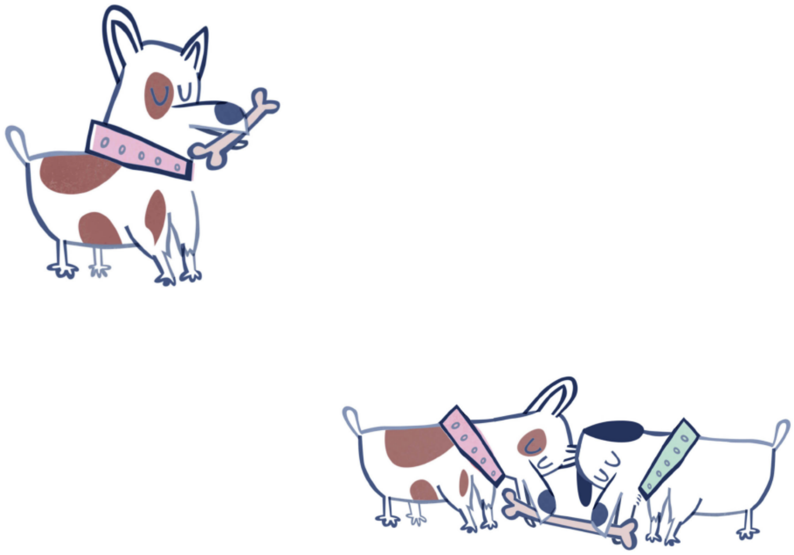

Sentence comprehension task of HIGA: picture set examples

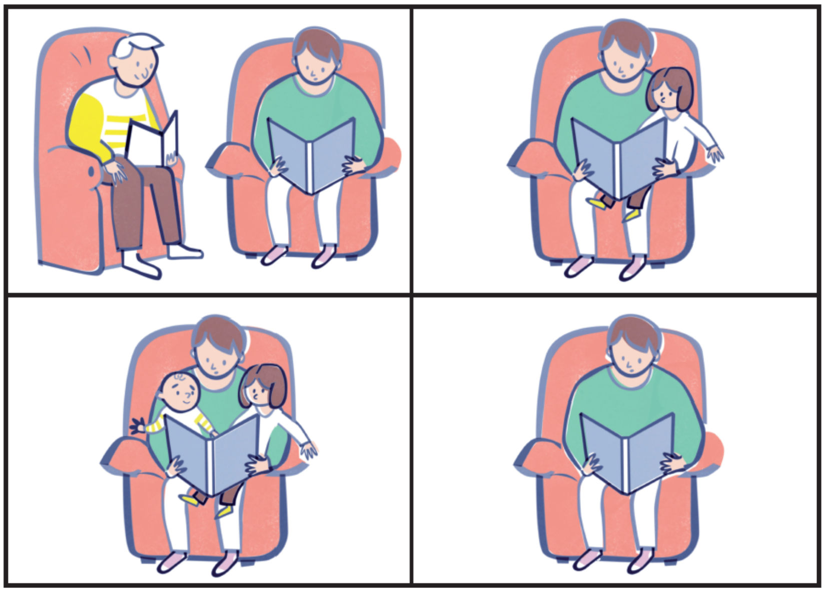

(© Illustrator C.Itssaga)

Supplementary material 6

'HEGA': Basque adaptation of PABIQ.

Haur Elebidunen Gurasoentzako galdetegia // Cuestionario para padres de un niño o una niña bilingüe // Questionnaire pour parents d'enfant bilingue

* Freely added questions, ** Added questions taken from the Basque MB-CDI parental questionnaire (Garcia et al., 2014)
**I. GENERAL INFORMATION (INFORMAZIO OROKORRA/ INFORMATION GENERALE/ DATOS GENERALES)**
1.0. Child's first and last name<br>1.01. Person completing the questionnaire<br>1.01'. In the previous question if you chose "Other", specify (grandparent, babysitter, etc.)<br>*1.02. Child's gender<br>**1.03. How many siblings does the child have?<br>**1.04. In what sibling position is the child (1st born, 2nd born, etc.)?<br>**1.05. How many people live together with the child? (CHILD INCLUDED)<br>1.06. Child's date of birth<br>1.07. Child's country of birth (+province+city)<br>1.08. Child's current country of residence (+province+city)<br>1.09. How long has the child lived in the Basque Country?<br>1.10. What languages does the child currently speak (more than just a few words)?<br>1.10'. In the previous question if you chose "Other", specify:<br>1.11. In which language do you think the child feels most comfortable?
**II. SCHOOL (*ESKOLA/ SCOLARITE/ ESCOLARIZACIÓN)**
*2.01. In which class is the child registered, and in which school?<br>*2.02. What school language model does the child follow?<br>*2.02'. In the previous question if you chose "Other", specify:<br>*2.03. Has the child changed language model since starting school? (for example, going from immersive 100% Basque to bilingual 50% Basque, or vice versa)<br>*2.04. Did the child skip a grade?<br>*2.05. Has the child repeated a grade?<br>*2.06. Does the child see a health professional inside or outside of school?<br>*2.06'. In the previous question if you chose "Other", specify:
**III. EARLY LANGUAGE HISTORY (HAURRAREN HISTORIA GOIZTIARRA/ HISTOIRE PRECOCE / HISTORIAL DEL NIÑO/DE LA NIÑA)**
3.01. At what age did your child produce his first word?<br>3.02. Around what age did your child put words together to make short sentences? (ex: more bread; more cake etc.)

3.03. Did you worry about his language?<br>Yes, before 3-4 years<br>*Yes, after 3-4 years<br>No, I don't think the child has language problems<br>3.04. Has your child had hearing problems or frequent ear infections?<br>3.04'. If you answered "yes" to the previous question, specify how often:<br>3.05. In general, BEFORE THE AGE OF 4, has your child been in contact with these languages:<br>[a. Basque]/[b. French]/[c. Spanish]/[d. Other]<br>3.05'. In the previous question, if you checked "Other", specify which language:<br>3.06. BEFORE THE AGE OF 4, did the child have contact with these languages [a. Basque]/[b. French]/[c. Spanish]/[d. Other] in these contexts?<br>[i. With his mother]<br>[ii. With his father]<br>[iii. With his brothers and sisters]<br>[iv. With his grandparents]<br>[v. With her babysitter]<br>[vi. With other adults]<br>[vii. At the nursery, daycare]<br>[viii. In kindergarden]<br>3.06a. At what age did the child begin to be in contact with<br>[a. Basque]/[b. French]/[c. Spanish]/[d. Other]
**IV. CURRENT SKILLS (HIZKUNTZA TREBETASUNAK/ HABILETES ACTUELLES/HABILIDADES ACTUALES)**
4.01. Compared to other children of the same age, how do you think the child expresses himself in …<br>[a. Basque]/[b. French]/[c. Spanish]/[d. Other]<br>4.02. Compared to other children of the same age, does the child have difficulty producing correct sentences in ...<br>[a. Basque]/[b. French]/[c. Spanish]/[d. Other]<br>4.03. Are you satisfied with the child's ability to express himself in...?<br>[a. Basque]/[b. French]/[c. Spanish]/[d. Other]<br>4.04. Does the child feel frustrated (or unhappy, annoyed) when he cannot communicate in…?<br>[a. Basque]/[b. French]/[c. Spanish]/[d. Other]<br>4.05. Do you think the child speaks X language like a child of the same age who only speaks this language X:<br>[a. Basque]/[b. French]/[c. Spanish]/[d. Other]
**V. LANGUAGE USE IN THE FAMILY**<br>**(HIZKUNTZEN ERABILERA/ COMPARAISON ENTRE LES LANGUES UTILISEES AU SEIN DE LA FAMILLE/ LENGUAS QUE USA LA FAMILIA)**
5.01a. Language(s) used between MOTHER and child

[a. Basque]/[b. French]/[c. Spanish]/[d. Other]
5.01'. In the previous question, if you checked "Other", specify in which language:
5.01b. Language(s) used between FATHER and child
[a. Basque]/[b. French]/[c. Spanish]/[d. Other]
5.01b'. In the previous question, if you checked "Other", specify in which language:
5.02. Language(s) used between ANOTHER ADULT who takes care of the child on a regular basis (grandparents, nanny, etc.) and the child
[a. Basque]/[b. French]/[c. Spanish]/[d. Other]
5.02'. In the previous question, if you checked "Other", specify in which language:
5.03. Language(s) used between the BROTHERS AND SISTERS and the child
[a. Basque]/[b. French]/[c. Spanish]/[d. Other]
5.03'. In the previous question, if you checked "Other", specify in which language:
*5.04. -"Badira unibertsitate anitz in Montpellier."- "Eta egin du à trois cent quinze, egin du le prix qui était sur le papier."- "Joan da Afrikarat, en coopération." Does the CHILD make this kind of production (2 languages in the same sentence) when speaking with someone else?
*5.04'. -If you answered "yes" to the previous question, please give an example here, if you can:
*5.05. -"Badira unibertsitate anitz in Montpellier."- "Eta egin du à trois cent quinze, egin du le prix qui était sur le papier."- "Joan da Afrikarat, en coopération." DO THE ADULTS IN HIS SURROUNDINGS make this kind of production (2 languages in the same sentence) when they speak with someone else?
*5.05'. -If you answered "yes" to the previous question, please give an example here, if you can:
**5.06. Please evaluate the percentage of Basque used with the child by those around him in general (parents, grandparents, teachers, guardians, etc.)

VI. LANGUAGE USE IN OTHER CONTEXTS (HIZKUNTZA AKTIBITATEAK/ LANGUES PARLEES DANS D'AUTRES CONTEXTES/ LENGUAS HABLADAS EN OTROS CONTEXTOS)

6.01. What language is used between your child and the friends with whom he plays regularly?
[a. Basque]/[b. French]/[c. Spanish]/[d. Other]
6.01'. In the previous question, if you checked "Other", specify in which language:
6.02. Which language(s) do family friends who come to your home regularly use?
[a. Basque]/[b. French]/[c. Spanish]/[d. Other]
6.02'. In the previous question, if you checked "Other", specify in which language:
6.03a. Does the child READ (books, magazines, comics, newspapers) every week in...
[a. Basque]/[b. French]/[c. Spanish]/[d. Other]
6.03a'. In the previous question, if you checked "Other", specify in which language:
*6.03b. To the child, DO YOU READ HIM (books, magazines, comics, newspapers) every week in...
[a. Basque]/[b. French]/[c. Spanish]/[d. Other]
6.03b'. In the previous question, if you checked "Other", specify in which language:
6.03c. Does the child WATCH TV or movies every week in...
[a. Basque]/[b. French]/[c. Spanish]/[d. Other]

6.03c'. In the previous question, if you checked "Other", specify in which language:<br>6.03d. Does the child TELL events or stories each week in ...<br>[a. Basque]/[b. French]/[c. Spanish]/[d. Other]<br>6.03d'. In the previous question, if you checked "Other", specify in which language:
**VII. INFORMATION ABOUT PARENTS**<br>**(GURASOEI BURUZKO INFORMAZIOA/ INFORMATIONS SUR LES PARENTS/ INFORMACION SOBRE LOS PADRES)**
7.01. In which country (city and province) was the MOTHER (1st guardian) of the child born?<br>7.01b. In which country (city and province) was the child's FATHER (2nd guardian) born?<br>7.02. If the child's parents are currently working, what language do they use at work?<br>7.02'. In the previous question, if you checked "Other(s)", specify in which language(s)<br>7.03. According to you, what is the level of the MOTHER (1st tutor) of the child in the following languages (tick the corresponding boxes)<br>[a. Basque]/[b. French]/[c. Spanish]/[d. Other]<br>7.03'. In the previous question, if you checked "Other(s)", specify in which language(s)<br>7.03b. In your opinion, what is the level of the PERE (2nd tutor) in the following languages (tick the corresponding boxes)<br>[a. Basque]/[b. French]/[c. Spanish]/[d. Other]<br>7.03b'. In the previous question, if you checked "Other(s)", specify in which language(s)<br>**7.04. Since when do the child's parents speak Basque? [Mother (or 1st guardian) of the child]<br>**7.04. Since when do the child's parents speak Basque? [Father (or 2nd guardian) of the child]<br>**7.05. Between the parents of the child, do you speak:<br>**7.06. Indicate the highest level of education of the parents of the child [Mother (or 1st guardian) of the child]<br>**7.06. Indicate the highest level of education of the parents of the child [Father (or 2nd guardian) of the child]
**VIII. DIFFICULTIES (ZAILTASUNAK/ DIFFICULTES/ DIFICULTADES)**
8.01-03. Tick if YES the child's entourage presents difficulties<br>[a. in reading and spelling]<br>[b. to understand others when they speak in the same mother tongue]<br>[c. to express themselves orally (pronunciation, form sentences, find the right word, etc.) in the mother tongue]
**IX. FREE COMMENTS (OHARRAK/COMENTARIOS/COMMENTAIRES)**<br>If you want to express ideas, write your comments here:

Supplementary material 7

Accepted and rejected answer types in the Object naming task (The target word is shown in capitals in brackets)

1	"ONGI"=1 = "GOOD"=1 considered as accepted answer types		
1	DET	adding a determiner	(ETXEA) etxe bat, etxea bat<br>(‘HOUSE’) a house, a the-house
2	FON	phonological change	(ETXEA) etxia<br>(‘HOUSE’ etʃea) /etʃia/
3	HIZK-FR, AZ	in French, and then autocorrection	(HARTZA) ours, hartza<br>(‘BEAR’) bear(FR), bear(B)
4<br>2	HIZK-FR,<br>FON<br>3	in French, and then autocorrection with phonological change	(HODEIA) nuage, (h) hodea<br>(odeia) /odea/<br>(‘CLOUD’) cloud(FR), cloud
5	I+ADJ	noun+adjective	(HARTZA) hartza xuria<br>(‘BEAR’) white bear
6	I+KASU	noun+case	(ETXEA) etxean<br>(‘HOUSE’) in the house
7	PL	Plural	(IPURDIA) ipurdiak<br>(‘BUTTOCK’) buttocks
8	SEM, AZ	semantically related, autocorrection	(KORAPILOA) soka, korapiloa<br>(‘KNOT’) rope, knot
9	SIN	synonymous	(GALTZERDIA) galtza<br>(‘SOCK’) sock
10	BESTE, AZ	uncategorizable, autocorrection	(XINAURRIA) ixaurria, xinaurria<br>(‘ANT’) nta, aunt
"BESTE"=0 = "OTHER"=0 considered as rejected answer types			
1	BESTE	uncategorizable	(GONA) zalipa<br>(‘SKIRT’) kisza
2	BESTE, HIZK-FR	uncategorizable, then in French	(IGELA) icona, crapaud<br>(‘TOAD’) pail, toad(FR)
3	BESTE, SEM	uncategorizable, then semantically related	(ILARGIA) lolo, (h) iguzkia<br>(‘MOON’) lolo, sun
4	HIZK-FR	In French	(HARTZA) le nours<br>(‘BEAR’) bear(FR)
5	HIZK-FR, BESTE	in French, then uncategorizable	(ESKORGA) brouette… erratza bat<br>(‘WHEELBARROW’)

			wheelbarrow(FR)… a broom
6	HIZK-FR, SEM	in French, then semantically related	(XINAURRIA)fourmi, (h) sagua<br>(‘AUNT’)aunt(FR), mouse
7	HIZK-FR+BESTE	in French and uncategorizable	(ILARGIA) une pomme<br>(‘MOON’) an apple(FR)
8	HIZK-FR+DET	in French determiner	(XINAURRIA)furmi bat<br>(‘AUNT’) an aunt(FR)
9	Hizk-FR+FON	in French + phonological change	(HARTZA)ous<br>(‘BEAR’) ber(FR)
10	HIZK-FR+PL	in French+plural	(LABEA) des fours<br>(‘OVEN’) ovens(FR)
11	HIZK-FR+SEM	in French+semantically related	(LABEA)four micro-ondes<br>(‘OVEN’)micro-waves(FR)
12	HIZK-FR+SEM+FON	in French+semantically related+phonological change	(LABEA) zofage<br>(‘OVEN’) feating(FR)
13	HIZK-GZ	In Spanish	(SASKIA) zesta bat<br>(‘A BASKET’) a basket(SP)
14	ONOM	onomatopoeia	(IGELA) boin boin<br>(‘FROG’) boin boin
15	SEM	semantically related	(BIBOLINA) gitarra<br>(‘VIOLIN’) guitar
16	SEM, BESTE	semantically related, and uncategorizable	(KORAPILOA) soka, floka<br>(‘KNOT’) rope, floka
17	SEM, HIZK-FR	semantically related, then in French	(KORAPILOA) zintura, nœud<br>(‘KNOT’) belt, knot(FR)
18	SEM, HIZK-FR+SEM	semantically related, then in French+ semantically related	(GONA) galtza, pantalon<br>(‘SKIRT’) pants(B), pants(FR)
19	SEM, SEM	semantically related, semantically related	(BIBOLINA) gitara, bateria<br>(‘VIOLIN’) guitar, battery
20	SEM+DET	semantically related+determiner	(BIBOLINA) gitarra bat<br>(‘VIOLIN’) a guitar
21	SEM+FON	semantically related/phonological	(GEREZIA)zakarra<br>(‘CHERRY’) affle

		change	
22	SEM+PL	semantically related+plural	(OPARIA) sorpresak<br>(‘PRESENT’) Suprises

Supplementary material 8

Pearson correlation analyses with the mother’s (self-rated) proficiency in Basque or French

	Basque		French	
	r	*p*	*r*	*p*
Current skills (max. 15 pts)	.528	< .001	.311	< .001
Age of first contact	-.491	< .001	-.263	< .001
Early language exposure ratio (before age 4)	.682	< .001	.427	< .001
Length of exposure	.269	< .001	.287	< .001
Language used at home (max. 16 pts)	.756	< .001	.393	< .001
Language richness (max. 16 pts)	.609	< .001	.375	< .001

Pearson correlation analyses with the father’s (self-rated) proficiency in Basque or French

	Basque		French	
	r	*p*	*r*	*p*
Current skills (max. 15 pts)	.445	< .001	.233	.002
Age of first contact	-.480	< .001	-.156	.039
Early language exposure ratio (before age 4)	.545	< .001	.193	.010
Length of exposure	.229	.002	.119	.115
Language used at home (max. 16 pts)	.651	< .001	.199	.007
Language richness (max. 16 pts)	.527	< .001	0.168	.023

Table S1 (supplementary material). Results on bilingualism variables, for Basque and French, according to schooling type (Basque only or Bilingual Basque/French)

	Basque					French				
	Ba only	Ba/Fr	*t*	*df*[a]	*p*	Ba only	Ba/Fr	*t*	*df*[a]	*p*
Age of first contact (months)	6.0 (13.3)	18.2 (18.1)	-4.821	171	< .001	3.6 (11.2)	0.0 (0.0)	--	--	--
Length of exposure to Basque or French (months)	73.4 (21.0)	71.4 (21.3)	0.552	171	.582	76.2 (20.5)	89.7 (11.3)	-4.281	174	< .001
Frequency of early exposure (1-4 scale)	3.3 (1.0)	2.3 (1.9)	5.821	182	< .001	2.9 (1.2)	3.8 (0.4)	-5.088	182	< .001
Early contacts total (max. 8 pts)	5.2 (1.9)	2.9 (1.5)	7.607	179	< .001	4.2 (1.8)	5.9 (1.6)	-5.729	177	< .001
Early language exposure ratio (before age 4)	55.4 (19.0)	30.4 (12.8)	8.484	182	< .001	43.4 (17.2)	66.5 (13.0)	-8.443	177	< .001
Language used at home (max. 16 pts)	10.4 (4.4)	4.3 (3.0)	8.733	182	< .001	8.8 (4.9)	14.4 (2.3)	-7.493	182	< .001
Language richness (max. 24 pts)	12.9 (4.6)	4.2 (2.5)	12.477	182	< .001	11.0 (6.6)	21.5 (3.0)	-10.681	182	< .001
Proficiency level (mother) (0-4 scale)	2.6 (1.2)	1.0 (1.1)	7.852	182	< .001	3.8 (0.4)	4.0 (0.2)	-2.872	180	.005
Proficiency level (father) (0-4 scale)	2.2 (1.4)	1.0 (1.4)	4.849	182	< .001	3.8 (0.5)	3.8 (0.6)	-0.333	180	.740
Current skills (max. 15 pts)	10.0 (3.4)	7.3 (3.7)	4.626	182	< .001	8.9 (3.7	11.8 (2.5)	-5.126	182	< .001

Key: Ba only = Basque only; Ba/Fr = Bilingual Basque/French

[a] *df* differed according to the number of parents who provided the requested information

Table S2 (supplementary material). Results on markers of early language development milestones (age of word and first sentence) and the non-risk index in Basque only and Bilingual Basque/French schools

	Basque only	Bilingual Basque/French	*t*	*df*[a]	*p*
Age of first word (0-6 scale)	5.1 (1.3)	5.3 (0.9)	-1.105	170	.271
Age of first sentence (0-6 scale)	5.1 (1.3)	5.5 (0.9)	-1.884	169	.061
Non-risk index (max. 23 pts)	20.5 (3.0)	21.5 (2.3)	-1.911	169	.058

Note: The following scale was used for estimating age of first word and age of first sentence: 0 = significantly delayed first word (> 24 months) and first sentence (> 36 months), 4 = sightly delayed first word (12-24 months) and first sentence (24-36 months), and 6 = no elayed first word (< 12 months) and first sentence (< 24 months)

[a] *df* differed according to the number of parents who provided the requested information

Figure 1 (supplementary material). Scattered plots and boxplots presenting the scores for each language task and how they relate to the variables investigated in this study.

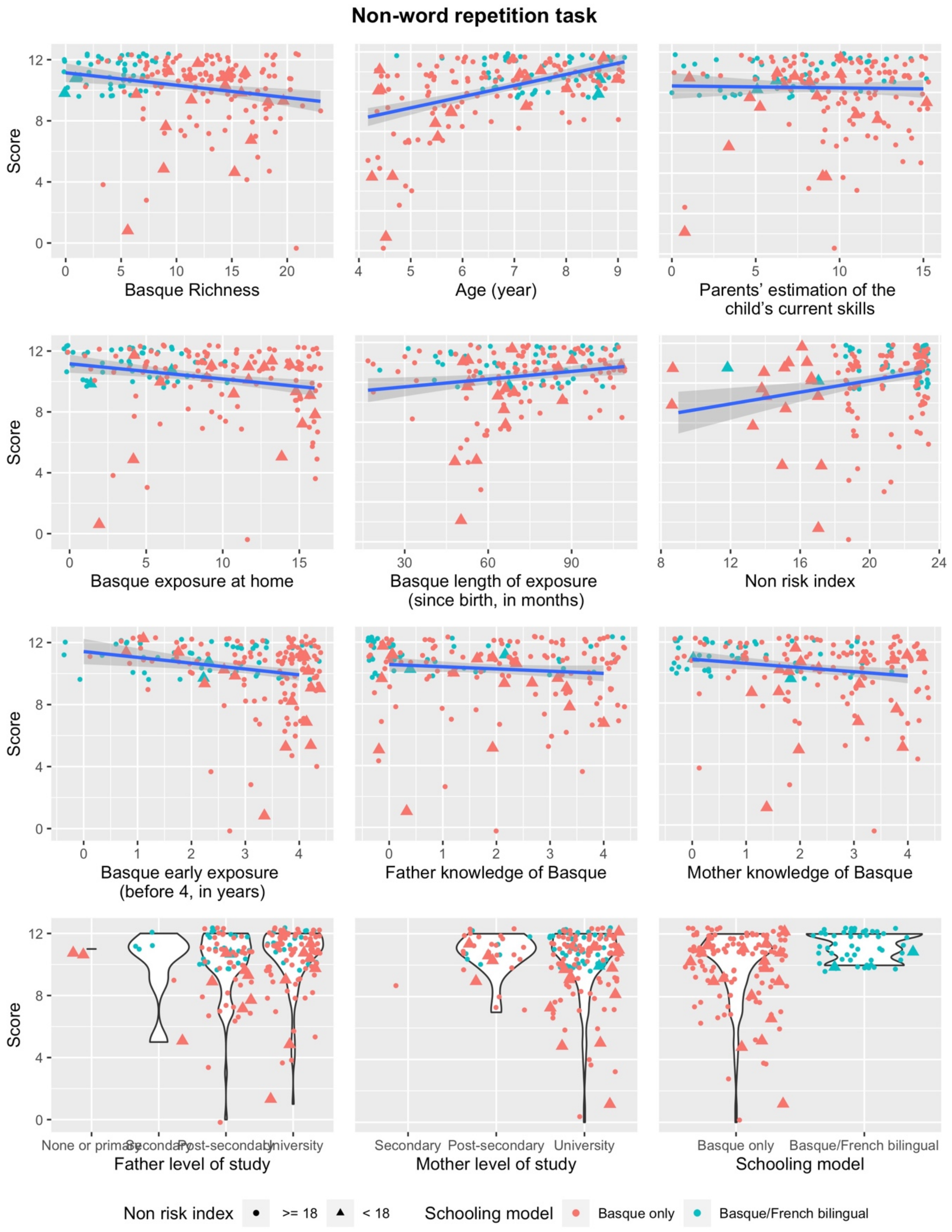
Non-word repetition task
Score
Basque Richness
Age (year)
Parents' estimation of the child's current skills
Basque exposure at home
Basque length of exposure (since birth, in months)
Non risk index
Basque early exposure (before 4, in years)
Father knowledge of Basque
Mother knowledge of Basque
None or primary
Secondary
Post-secondary
University
Father level of study
Mother level of study
Basque only
Basque/French bilingual
Schooling model
Non risk index
>= 18
< 18

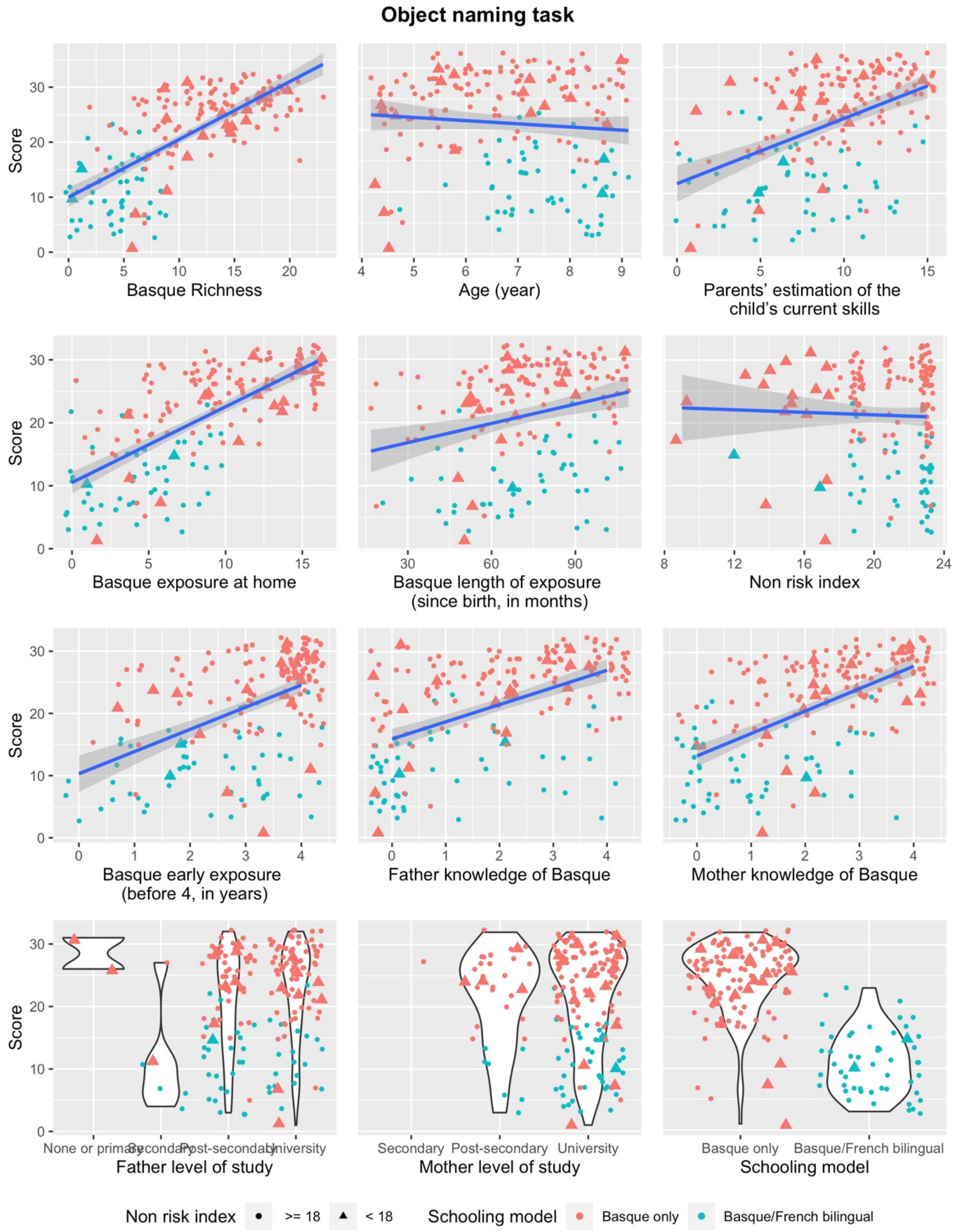
Object naming task
Score
Basque Richness
Age (year)
Parents' estimation of the child's current skills
Basque exposure at home
Basque length of exposure (since birth, in months)
Non risk index
Basque early exposure (before 4, in years)
Father knowledge of Basque
Mother knowledge of Basque
None or primary
Secondary
Post-secondary
University
Father level of study
Mother level of study
Basque only
Basque/French bilingual
Schooling model
Non risk index
>= 18
< 18
Schooling model
Basque only
Basque/French bilingual

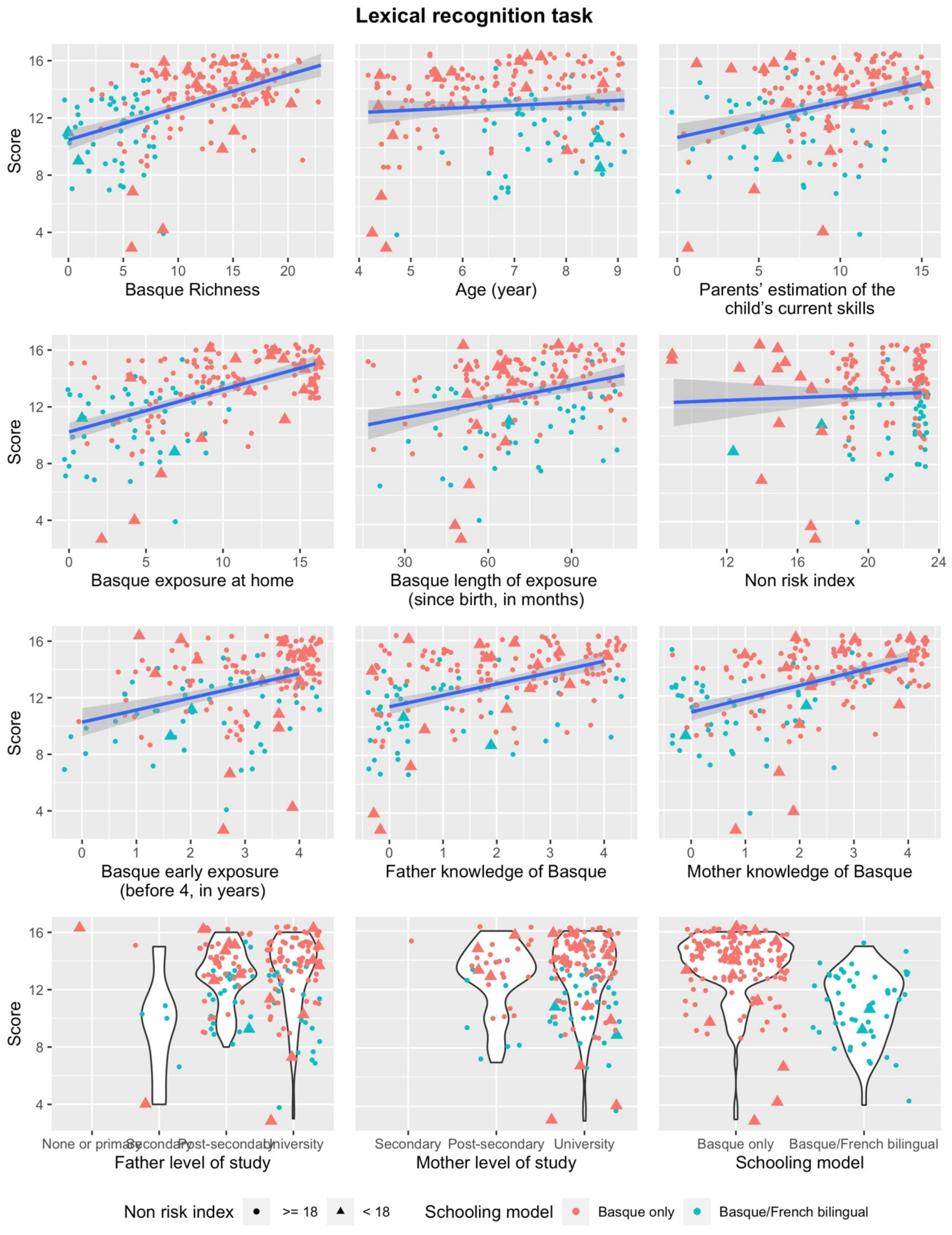
Lexical recognition task
Score
Basque Richness
Age (year)
Parents' estimation of the child's current skills
Basque exposure at home
Basque length of exposure (since birth, in months)
Non risk index
Basque early exposure (before 4, in years)
Father knowledge of Basque
Mother knowledge of Basque
None or primary
Secondary
Post-secondary
University
Father level of study
Mother level of study
Basque only
Basque/French bilingual
Schooling model
Non risk index
>= 18
< 18
Schooling model
Basque only
Basque/French bilingual

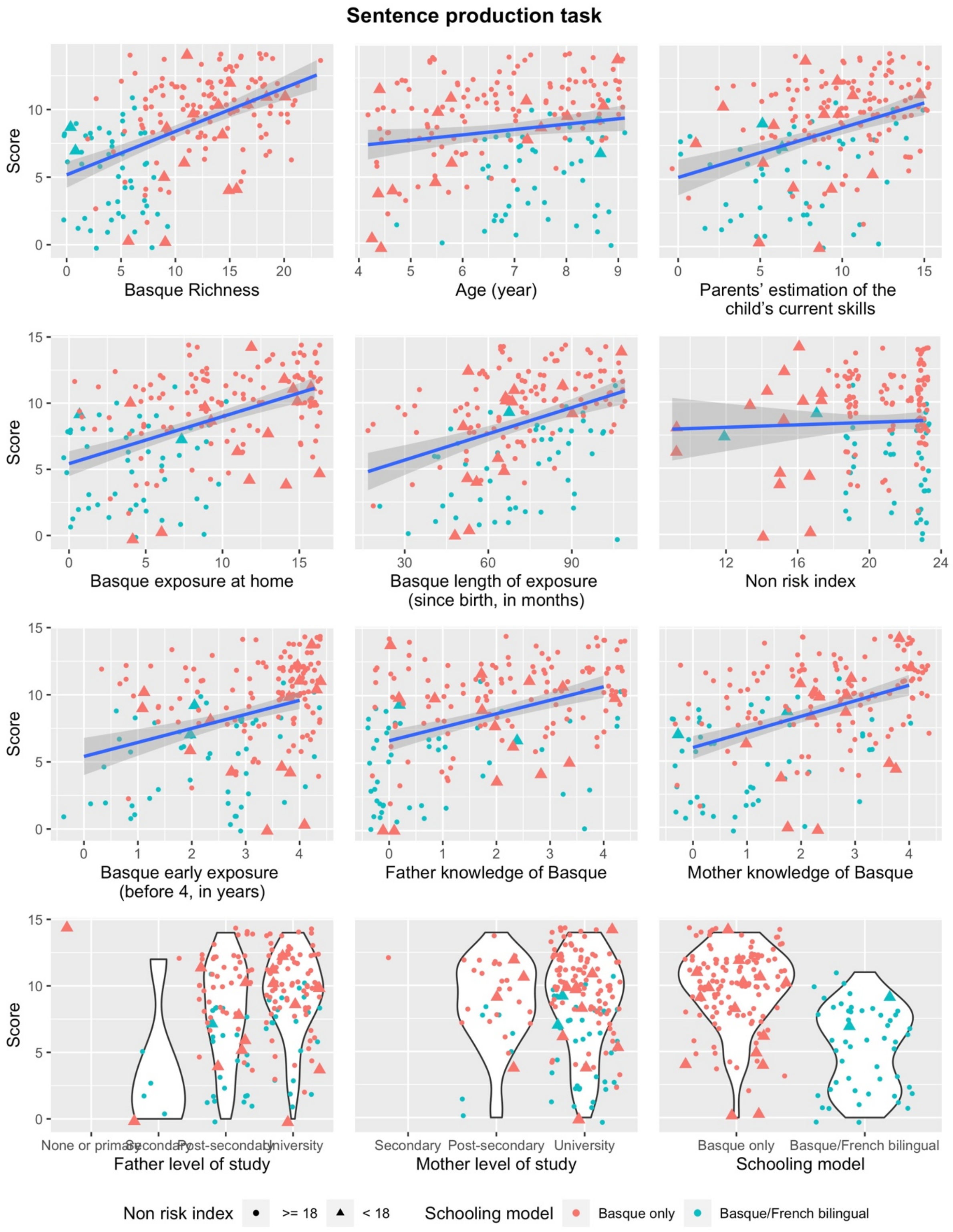
Sentence production task
Score
Basque Richness
Age (year)
Parents' estimation of the child's current skills
Basque exposure at home
Basque length of exposure (since birth, in months)
Non risk index
Basque early exposure (before 4, in years)
Father knowledge of Basque
Mother knowledge of Basque
None or primary
Secondary
Post-secondary
University
Father level of study
Mother level of study
Basque only
Basque/French bilingual
Schooling model
Non risk index
>= 18
< 18

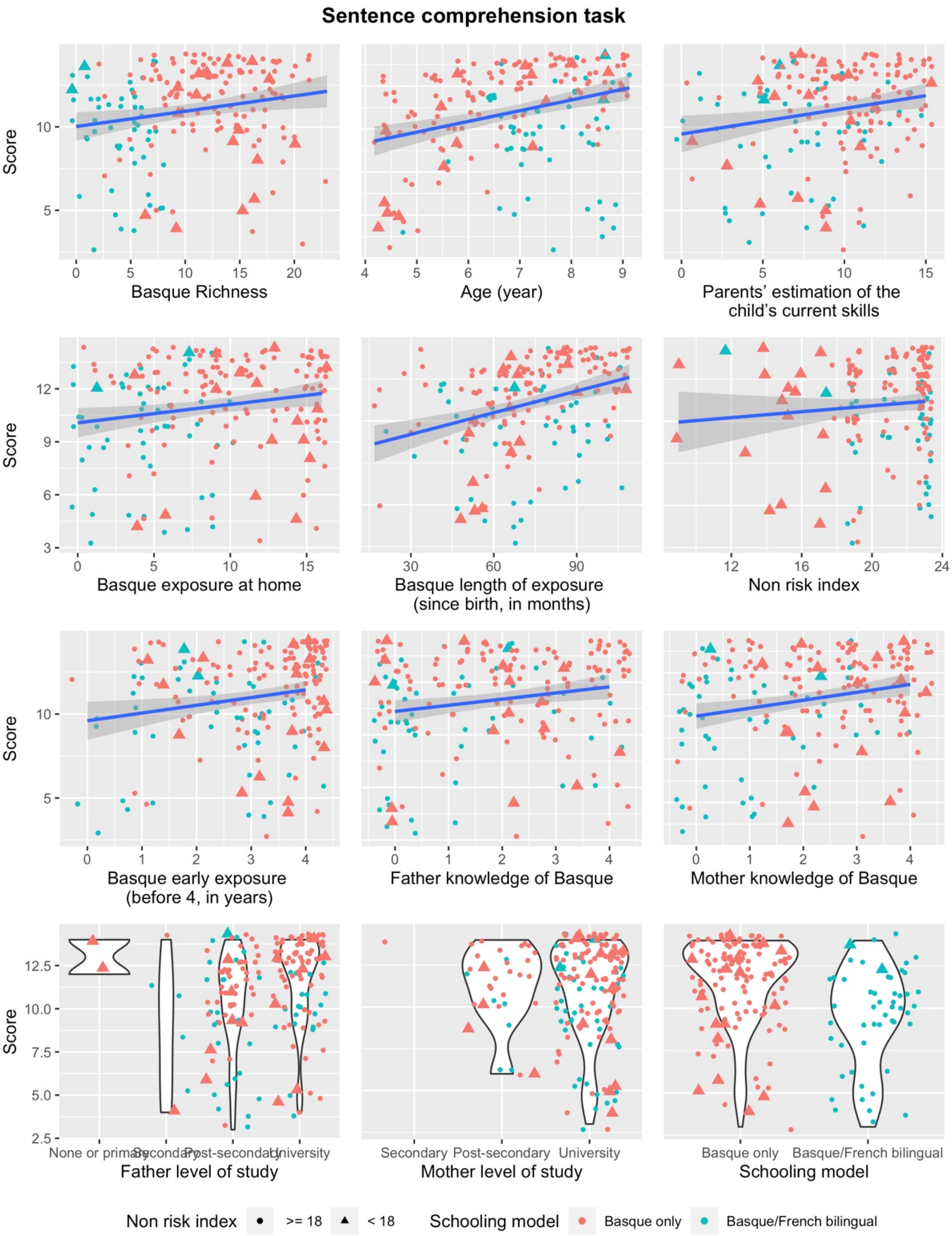
Sentence comprehension task
Score
Basque Richness
Age (year)
Parents' estimation of the child's current skills
Basque exposure at home
Basque length of exposure (since birth, in months)
Non risk index
Basque early exposure (before 4, in years)
Father knowledge of Basque
Mother knowledge of Basque
None or primary
Secondary
Post-secondary
University
Father level of study
Mother level of study
Basque only
Basque/French bilingual
Schooling model
Non risk index
>= 18
< 18
Schooling model
Basque only
Basque/French bilingual
